# Reframing ontology fact acquisition during large language model fine-tuning as a time-to-event process

**DOI:** 10.3389/frai.2026.1805890

**Published:** 2026-06-22

**Authors:** Daniel B. Hier, Tayo Obafemi-Ajayi

**Affiliations:** 1 Department of Neurology and Rehabilitation, University of Illinois at Chicago, Chicago, IL, United States; 2 Engineering Program, Missouri State University, Springfield, MO, United States

**Keywords:** Cox proportional hazards model, fine-tuning, Gene Ontology, Human Phenotype Ontology, Kaplan–Meier estimator, Llama-3.1 Instruct

## Abstract

**Introduction:**

Large language models can be fine-tuned to improve retrieval of biomedical facts that are not reliably accessible after pretraining. We used ontology term-identifier mappings from the Human Phenotype Ontology (HPO) and Gene Ontology (GO) as structured biomedical facts to study fact acquisition during fine-tuning.

**Methods:**

Each ontology fact consisted of a term and its corresponding machine-readable identifier. We reframed fact acquisition as a discrete time-to-event process indexed by training epoch. Llama-3.1-8B Instruct was fine-tuned on HPO and GO ontology facts. Deterministic retrieval accuracy was assessed at baseline and after each fine-tuning epoch. Repeated stochastic decoding at baseline was used to probe for latent parametric support. Baseline-incorrect facts that were recovered at least once under stochastic decoding were classified as *latent-knowledge-positive*; facts not recovered under stochastic decoding were classified as *latent-knowledge-negative*. We distinguished trained-fact acquisition, defined for facts included in the fine-tuning set, from untrained-fact acquisition, defined for facts withheld from training. Fact loss was defined as the first transition from correct to incorrect retrieval among facts that were correct in the base model. Kaplan-Meier estimators were used to construct fact-acquisition and fact-loss curves over training epochs, and Cox proportional hazards models were used to identify predictors of acquisition rate.

**Results:**

At baseline, the model correctly retrieved 1.1% (9/800) of HPO facts and 5.6% (44/802) of GO facts. After 20 epochs of supervised fine-tuning, correct retrieval increased to 71.9% (575/800) for HPO-trained facts, 61.8% (248/401) for GO-trained facts, and 11.2% (45/401) for GO-untrained facts. Latent-knowledge-positive facts were acquired more efficiently than latent-knowledge-negative facts. Corpus-based fact support in the biomedical literature had smaller positive effects on fact acquisition. Untrained-fact acquisition for withheld GO facts was uncommon, occurring in 5.8% (22/378) of baseline-incorrect withheld facts, but was more likely for latent-knowledge-positive facts. Among GO facts that were correct at baseline, fact loss was more frequent for untrained facts than for trained facts, suggesting that continued training exposure was associated with greater retention of already-correct mappings.

**Discussion:**

Ontology facts provide a useful experimental model for studying fact acquisition during large language model fine-tuning. A time-to-event framework reveals not only whether facts are acquired, but also when they are acquired and whether they are later lost. The findings suggest that pretraining-derived latent knowledge, detectable through stochastic decoding, influences the rate and stability of fact acquisition during fine-tuning. This framework may help evaluate fine-tuning strategies, curriculum design, and the durability of learned biomedical facts.

## 1 Introduction

Although originally designed as text generators, large language models are now widely used as repositories of facts that can be queried on demand (Wang et al., 2023; Mousavi et al., 2026). This use is limited by knowledge gaps. Because these models are trained primarily on large text corpora rather than curated fact databases (Ovadia et al., 2024), the facts encoded in their parameters reflect the uneven distribution of information in their training data. Frequently encountered facts are more likely to be encoded strongly and retrieved reliably, whereas rare facts are harder to retrieve (Kandpal et al., 2023).

Fine-tuning is often used to improve the retrieval of factual knowledge encoded in model parameters, either by strengthening weakly represented associations or by making previously inaccessible associations reliably retrievable (Ghosal et al., 2024). Most studies of fact acquisition during fine-tuning frame success as the endpoint: a fact is either acquired or not. Less attention has been paid to the dynamics of acquisition: how rapidly different facts are learned and which facts are lost during training. Similarly, relatively little work has examined how measurable properties of the fact, the training process, or the model influence the rate and durability of fact acquisition.

In this study, we use ontology term–identifier mappings as a structured fact acquisition task. Each fact consists of a biomedical ontology term and its corresponding machine-readable identifier, such as *ataxia* → HP:0001251 in the Human Phenotype Ontology (HPO) or *nucleus* → GO:0005634 in the Gene Ontology (GO). These mappings are numerous, structurally comparable, objectively scorable, and incompletely retrieved before task-specific fine-tuning (Do et al., 2025a,b; Hier et al., 2025b; Pericharla et al., 2026). The same query format can be repeated across hundreds of facts, allowing fact-level learning trajectories to be measured directly.

Two classes of predictors are especially relevant to factual acquisition during fine-tuning. The first is *latent parametric support*: the extent to which a fact is already recoverable from the model before fine-tuning, even if it is not retrieved under deterministic decoding. The second is *corpus-based fact support*: the extent to which the fact, or its components, are represented in biomedical text. Because neither latent parametric support nor true pretraining exposure can usually be measured directly, both require operational probes. In this study, we use repeated stochastic decoding at baseline as a probe of latent parametric support, and curated annotation counts and PubMed Central term and identifier frequencies as external proxies for corpus-based fact support.

Prior work has shown that factual knowledge in LLMs is dynamic and can be strengthened, altered, generalized, or lost during pretraining and subsequent training (Lu et al., 2024; Chang et al., 2024; Speicher et al., 2024; Tirumala et al., 2022; Li et al., 2020). For example, Chang et al. (2024) analyze factual knowledge acquisition *during pretraining* using injected fictional knowledge and log-probability trajectories, distinguishing memorization, semantic generalization, compositional generalization, and forgetting. Related work has shown that fact popularity in pretraining corpora influences whether facts become encoded in model parameters (Kandpal et al., 2023; Ghosal et al., 2024; Hier et al., 2025a,b; Bombieri et al., 2025). Stochastic-decoding studies further suggest that some facts present in the model's weights may be inaccessible under deterministic decoding but recoverable through sampling, a phenomenon sometimes described as *hidden knowledge* (Gekhman et al., 2025). Together, these findings suggest that facts differ not only in whether they are represented, but also in how readily they can be retrieved and reinforced.

Here, we introduce a time-to-event framework for characterizing fact acquisition and loss during fine-tuning. Specifically, we record the epoch at which a trained fact first becomes correct, an untrained fact first becomes retrievable, or a previously correct fact is lost. This framing allows Kaplan–Meier estimators to describe the accumulation and loss of retrievable facts over epochs and Cox proportional hazards models to quantify how covariates such as latent parametric support and corpus-based fact support influence acquisition rates.

We apply this framework to ontology facts from HPO and GO. HPO serves as the testbed for modeling *trained-fact acquisition*, because all sampled HPO mappings are included in the fine-tuning set. GO serves as a complementary testbed because sampled GO mappings are split into trained and untrained sets, allowing us to evaluate both *untrained-fact acquisition* among mappings excluded from fine-tuning and *fact loss* among mappings that were correct at baseline. Fine-tuning epochs provide a natural timeline: an event occurs when a trained mapping first becomes correct, when a held-out mapping first emerges despite exclusion from training, or when a baseline-correct mapping is no longer retrieved correctly.

We hypothesize that facts with latent parametric support in the base model are acquired more rapidly when directly trained, are more likely to emerge despite being held out, and are less vulnerable to loss after correct retrieval (Gekhman et al., 2025; Hier et al., 2025a; Pletenev et al., 2025). We further test whether external proxies for corpus-based fact support, including PubMed Central term and identifier frequencies and curated annotation counts, are associated with these event-time outcomes.

The remainder of this paper is structured as follows. Section 2 describes the datasets, fine-tuning procedure, and methods for measuring *trained-fact acquisition, untrained-fact acquisition*, and *fact loss*. Section 3 presents results across HPO and GO, including fact-level acquisition rates and predictors of acquisition and loss. Section 4 discusses the implications for terminology-aware fine-tuning and outlines limitations and future directions.

## 2 Methods

### 2.1 Terminology

**Figure 1:**
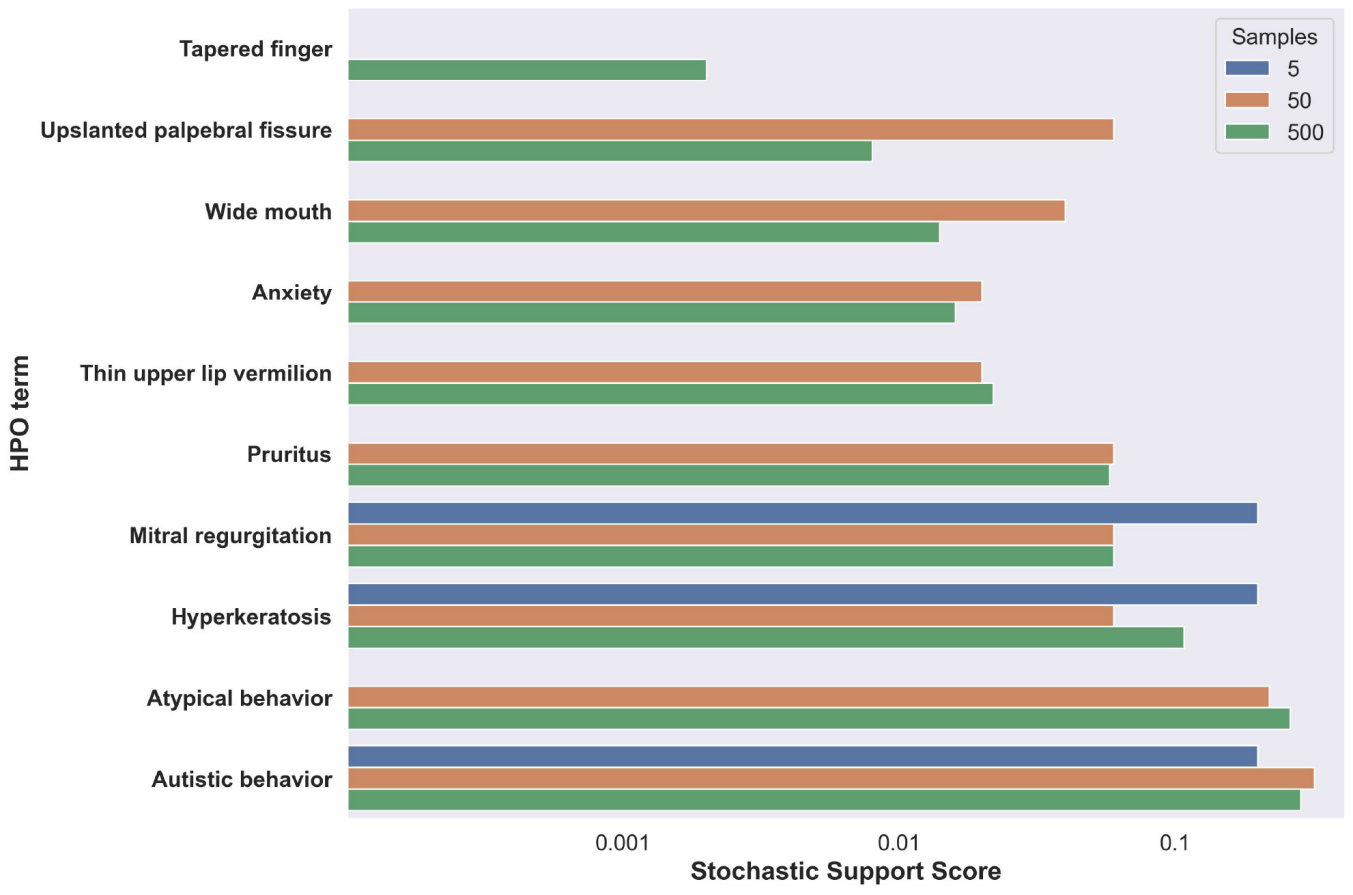
Sensitivity analysis of HPO stochastic support score estimates across sample sizes of 5, 50, and 500 trials. All HPO terms were scored incorrectly under deterministic decoding. The stochastic support score is the proportion of samples correct under stochastic decoding. The *y*-axis is log-scaled. Except for the term *Tapered finger*, 50 samples (orange bars) were as sensitive to latent knowledge as 500 samples (green bars).

In this study, we evaluated the acquisition and loss of ontology term–identifier mappings during large language model fine-tuning. We refer to each term–identifier mapping as an *ontology fact*. Because several related terms are used inconsistently in the literature, we use the following operational definitions:

- *Trained facts* are ontology facts included in the fine-tuning set. *Untrained facts* are ontology facts withheld from the fine-tuning set.
- *Fact acquisition* is the first transition from incorrect retrieval at baseline to correct retrieval at a subsequent fine-tuning epoch. This outcome may occur for both trained and untrained facts.
- *Fact loss* is the first transition from correct retrieval at baseline to incorrect retrieval at a subsequent fine-tuning epoch.
- *Deterministic decoding*, or *argmax decoding*, refers to selecting the highest-probability next token at each generation step. Unless otherwise specified, accuracy in this study refers to deterministic accuracy, defined as exact retrieval of the correct ontology identifier under deterministic decoding.
- *Stochastic decoding* refers to repeated sampling from the model's output distribution at an elevated temperature. We used stochastic decoding before fine-tuning to probe for term–identifier associations that were not retrieved under deterministic decoding but could nevertheless be generated by the model (Gekhman et al., 2025). *Stochastic support* was defined as the fraction of repeated stochastic queries that produced the correct ontology identifier; in this study, it was calculated from 50 stochastic responses per term–identifier mapping. We interpret stochastic support as an operational proxy for latent parametric support: the degree to which a term–identifier association is recoverable from the model before fine-tuning.
- *Latent-knowledge-positive facts* are baseline-incorrect facts for which the correct identifier was produced at least once in 50 stochastic samples before fine-tuning. *Latent-knowledge-negative facts* are baseline-incorrect facts for which the correct identifier was not produced in any of the 50 stochastic samples. The 50-sample threshold was chosen as a compromise between sensitivity and practicality; sensitivity analyses for 10 terms are shown in Figure 1.
- *Corpus-based fact support*, also called *fact popularity*, refers to the extent to which an ontology fact, or its components, is represented in biomedical text or curated biomedical annotations. Because true pretraining exposure cannot usually be measured directly, we used external proxies for corpus support: ontology annotation counts and counts of term and identifier occurrences in PubMed Central.

### 2.2 Datasets

We used facts from the *Human Phenotype Ontology* (HPO) and the *Gene Ontology* (GO) as structured factual units for fine-tuning (Robinson et al., 2015; Ashburner et al., 2000). Each ontology fact consisted of a single ontology term and its corresponding machine-readable identifier, such as *ataxia* → HP:0001251 in HPO or *nucleus* → GO:0005634 in GO. These mappings are widely used across NLP and ontology-based applications in biomedicine and provide objectively scorable examples of biomedical factual retrieval.

The full HPO contains 18,961 concepts. From this ontology, we sampled 800 facts, stratified to include both frequently occurring terms and long-tail terms that appear rarely in biomedical text. All 800 HPO facts were included in the fine-tuning set as *trained facts*. GO contains 39,354 terms across three hierarchies: biological process, molecular function, and cellular component. We sampled 802 GO facts using an analogous frequency-stratified strategy. GO facts were divided into a training set of *trained facts* and a held-out evaluation set of *untrained facts*. The trained and untrained GO sets were balanced with respect to the key covariates of interest: PubMed Central identifier counts, PubMed Central term counts, and GO annotation counts (Table 1). Terms that never appeared in curated literature annotations—approximately 40% of HPO and 54% of GO terms (Hier et al., 2025b)—were excluded to ensure that the tested facts had at least minimal grounding in curated biomedical annotations.

**Table 1:** Baseline balance of trained and untrained GO facts.

Feature	Untrained (*n* = 401)	SD	Trained (*n* = 401)	SD	*t*-statistic	*p*-value
Baseline covariates						
Correct at baseline	5.7%	22.3%	5.3%	22.3%	–0.31	0.76
Stochastic support score	0.03	0.13	0.04	0.15	0.68	0.50
GO annotations	215	1,677	209	1,906	–0.05	0.96
PMC ID count	26.51	87.12	24.86	85.85	–0.27	0.79
PMC term count	15,862	130,245	11,702	70,379	–0.56	0.57
Length (characters)	24.9	11.9	24.5	12.0	0.46	0.64
Post-fine-tuning outcome						
Correct after fine-tuning	11.2%	31.6%	61.8%	48.6%	17.48	<0.001

*The table compares trained and untrained GO facts to assess baseline balance before fine-tuning. Stochastic support score is the proportion of stochastic responses that produced the correct identifier for each fact at baseline using temperature 1.0. For baseline-incorrect facts, nonzero stochastic support was used to define latent-knowledge-positive status. Baseline covariates were similar between groups, whereas post-fine-tuning accuracy was substantially higher among trained facts than untrained facts*.

*Corpus-based fact support*, or *fact popularity*, was estimated using two complementary sources that approximate the biomedical salience of each ontology fact (Kandpal et al., 2023).

1. *Text-frequency support*: identifier and term frequencies were estimated from PubMed Central (PMC) full-text records (Hier et al., 2025a; Do et al., 2025a,b). Each ontology term label and each ontology identifier were queried separately using the PMC API (https://eutils.ncbi.nlm.nih.gov/entrez/eutils/), and the number of matching records was recorded as the corresponding term-frequency or identifier-frequency count.
2. *Curated annotation support*: annotation counts were derived from ontology annotation files. For HPO, we used the phenotype.hpoa annotation file, which links diseases to HPO identifiers and supporting PubMed references and is distributed by the Human Phenotype Ontology project (https://hpo.jax.org/data/annotations). For each HPO identifier, we counted the number of disease annotations in which that identifier appeared. For GO, we used the goa_human.gaf annotation file, which links human genes or proteins to GO identifiers and supporting references and is distributed through the Gene Ontology annotation downloads (https://current.geneontology.org/products/pages/downloads.html). For each GO identifier, we counted the number of human gene/protein annotations in which that identifier appeared.

These measures were treated as external proxies for corpus-based fact support (Ni et al., 2025), not as direct measurements of the model's pretraining distribution. The supporting PubMed references in the HPO and GO annotation files identify literature used to justify curated annotations, but they do not necessarily imply that the publication explicitly contains the ontology identifier or exact term label. Thus, PMC term and identifier counts provide text-frequency proxies, whereas curated annotation counts provide biocuration-frequency proxies.

### 2.3 Fine-tuning

We fine-tuned the Llama-3.1-8B Instruct model on ontology facts using a parameter-efficient approach. Fine-tuning was hosted by Together AI (https://www.together.ai/). For HPO, all 800 sampled facts were included in training as *trained facts*. To increase robustness to prompt phrasing, we generated five paraphrased prompts for each fact, yielding 4,000 training instances per epoch (Table 2).

**Table 2:** Example paraphrased training prompts for one HPO term–identifier pair.

No.	User prompt	Assistant response
1	What is the Human Phenotype Ontology ID for the term: “Hyperpigmentation of the skin”?	HP:0000953
2	Please provide the ontology ID for “Hyperpigmentation of the skin” from the HPO.	HP:0000953
3	Find the correct HPO identifier for the phenotype: Hyperpigmentation of the skin.	HP:0000953
4	Which Human Phenotype Ontology ID corresponds to the clinical term “Hyperpigmentation of the skin”?	HP:0000953
5	Give me the standardized HPO code for this term: Hyperpigmentation of the skin.	HP:0000953

*Each term–identifier pair was represented by five paraphrased instruction templates during supervised fine-tuning. The target assistant response was the canonical ontology identifier only*.

For GO, the 802 sampled facts were split evenly into a training set of 401 *trained facts* and a withheld evaluation set of 401 *untrained facts*. The withheld facts were not presented to the model during fine-tuning, enabling a measurement of *untrained-fact acquisition*. Trained and untrained facts were balanced on the covariates of interest (Table 3). For each term-identifier pair in the training set, five variant prompt phrasings were used (Table 2).

**Table 3:** Covariates by fact-loss status during fine-tuning.

Covariate	Fact loss (*n* = 24)	No fact loss (*n* = 20)	*t*-value	*p*-value
Trained (binary)	0.10 (0.30)	0.83 (0.39)	7.02	<0.01
Stochastic sampling accuracy	0.38 (0.30)	0.60 (0.29)	2.48	0.02
GO ID PMC count	175 (245)	256 (259)	1.07	0.29
GO term PMC count	66,885 (127,700)	168,536 (254,006)	1.70	0.10
GO annotations	1,760 (4,654)	4,069 (8,797)	1.10	0.28

*Values are mean (SD). Count variables are long-tailed and are shown descriptively; statistical comparisons should be interpreted cautiously because of the small sample size and skewed distributions. Trained status and stochastic sampling accuracy was lower in the fact loss group*.

Fine-tuning was performed using Low-Rank Adaptation (LoRA) (Hu et al., 2021) with Together AI's default all-linear configuration, which applies LoRA adapters to all supported linear transformer modules for this architecture. An analogous procedure was used for GO facts. Unless otherwise stated, the LoRA configuration used rank *r* = 64 and scaling α = 128. Optimization used a cosine learning-rate scheduler with initial learning rate 1 × 10^−5^, no warm-up, batch size 32, maximum gradient norm 1, and zero weight decay. The Together AI job metadata reported scheduler_cycles = 0.5; this parameter was retained as part of the platform scheduler configuration and was not manually varied. The train-on-inputs option was set to auto, allowing the platform to apply its default input-masking behavior. Fine-tuned checkpoints were generated at each epoch from 1 through 20. Epoch 0 corresponded to the Llama-3.1-8B Instruct model before LoRA fine-tuning.

### 2.4 Model assessment and identifier scoring

All fine-tuned model evaluations were performed locally using checkpoint artifacts downloaded from Together AI at each epoch, including merged safetensors shards, tokenizer files, configuration files, and chat templates. This ensured consistent inference conditions across all evaluations. Models were loaded with Hugging Face AutoTokenizer and AutoModelForCausalLM and run in evaluation mode. Accuracy was assessed using deterministic decoding (greedy generation) with do_sample = False, temperature = 0, top_p = 1.0, top_k = 0, repetition penalty = 1.0, and a maximum of 8 generated tokens. A fixed random seed was set for reproducibility. For each HPO term, the model was prompted with: What is the HPO ID for term? Return only the code with no explanation. A sensitivity analysis using alternative but semantically similar HPO evaluation prompts yielded comparable baseline retrieval results (Table 4).

What is the HPO ID for term? Return only the code with no explanation.

**Table 4:** Base-model sensitivity analysis for the evaluation prompt.

Prompt ID	Terms	Repeats per term	Accuracy	Prompt text
1	100	5	0.10	What is the HPO ID for HPO Term: [term]? Return only the HPO ID with no explanation.
2	100	5	0.06	Give the Human Phenotype Ontology identifier for the HPO term [term]. Return only the HPO ID.
3	100	5	0.08	Find the correct HPO ID for this HPO term: [term]. Return only the HPO identifier.
4	100	5	0.07	Which Human Phenotype Ontology ID corresponds to the HPO term [term]? Answer with the HPO ID only.
5	100	5	0.06	Return the HPO ID for the HPO term [term]. Do not include any explanation.

*Prompt ID 1 was used for the primary accuracy analyses. Five semantically similar prompts were tested on the unfine-tuned Llama-3.1-8B Instruct model using 100 HPO terms with five repeated deterministic evaluations per term for each prompt (500 trials per prompt; temperature = 0.0). This analysis was performed on the unfine-tuned base model and was intended to assess whether baseline HPO identifier retrieval was highly dependent on minor prompt wording changes. It was not a comprehensive prompt-engineering study and did not evaluate prompt sensitivity across all fine-tuned epoch checkpoints*.

An analogous procedure was used for GO facts.

Model outputs were parsed using a regular expression to extract identifiers of the form HP:0000000, HPO:0000000, GO:0000000, or analogous canonical ontology-code formats. Extracted identifiers were normalized to the canonical seven-digit format via zero-padding. A response was scored as correct only if the normalized identifier exactly matched the reference. Outputs containing additional text were accepted if a valid identifier could be extracted, but correctness still required an exact match. Review of the saved raw output logs did not identify malformed but recoverable identifier formats, such as alternative separators or spacing variants, as a meaningful source of scoring error. When the model produced an ontology identifier, outputs conformed to the expected canonical format, such as HP:0001251 or GO:0005634.

Although the Llama-3.1-8B tokenizer decomposes ontology identifiers into prefix and numeric subtokens (e.g., splitting the seven-digit code into segments such as 123-456-7), all primary analyses used strict complete-identifier matching. Prior work has shown that partial subtoken correctness can provide a graded signal of model knowledge (Hier et al., 2025a), but this study focuses on acquisition and retention of full identifiers as the outcome of interest.

### 2.5 Data analysis

We modeled fine-tuning as a discrete, epoch-indexed time-to-event process. For each ontology fact, the acquisition event was defined as the first fine-tuning epoch at which the model produced the correct identifier for the corresponding term. Standard survival quantities were used, including the survival function *S*(*t*), cumulative hazard *H*(*t*), and hazard function *h*(*t*) (Kaplan and Meier, 1958; Collett, 2023; Kleinbaum and Klein, 2020). We characterized fact acquisition using Equation 1:


$$\begin{array}{c} F\left(t\right)=1-S\left(t\right) \end{array}$$ (1)

Kaplan–Meier estimates of *S*(*t*) were used to derive the fact acquisition curve in Equation 1. Facts already retrieved correctly by the base model at epoch 0 were right-censored at baseline for acquisition analyses. Confidence intervals used Greenwood's formula with log–log transformation and were computed using the lifelines library (version 0.30.0).

Predictors of fact acquisition rate were assessed using Cox proportional hazards models (Cox, 1972), yielding adjusted hazard ratios (HRs) with 95% confidence intervals. Continuous covariates were standardized; binary covariates were left unscaled. Four covariates were included: latent-knowledge status and three corpus-support measures: (i) identifier frequency in PubMed Central, (ii) term frequency in PubMed Central, and (iii) curated annotation count (Hill et al., 2008). Corpus-support measures were Laplace-smoothed and log_10_-transformed before model fitting.

Because event times were observed at discrete epochs and many facts could be acquired at the same epoch, tied event times were handled using Efron's approximation in the Cox models. Scaled Schoenfeld residuals were calculated for all Cox proportional hazards models to evaluate the proportional hazards assumption and to assess evidence of non-proportionality over the fine-tuning period (In and Lee, 2019). Analyses were implemented in Python version 3.10.14 (Python Software Foundation, Wilmington, DE, USA) using lifelines, numpy, pandas, matplotlib, and scipy.

*Fact acquisition for untrained facts* was assessed using the 401 GO facts that were withheld from fine-tuning. Twenty-three withheld facts that were retrieved correctly at baseline (epoch 0) were excluded from this acquisition analysis, leaving 378 baseline-incorrect *untrained facts*. These facts were evaluated across fine-tuned models produced after each training epoch (epochs 1–20), while fine-tuning was performed only on the 401 *trained facts*. For each untrained fact, the first transition from incorrect retrieval to correct identifier retrieval was recorded as an *untrained-fact acquisition* event occurring at that epoch. Kaplan–Meier estimators and Cox proportional hazards models were used to characterize acquisition rates and associated predictors, following standard survival-analysis procedures implemented in the lifelines Python library (version 0.30.0).

*Fact loss* was assessed among all GO facts that were retrieved correctly at baseline (epoch 0). This cohort included 44 baseline-correct facts: 21 trained facts and 23 untrained facts. These initially correct facts were re-evaluated across fine-tuned models produced after each training epoch (epochs 1–20), while fine-tuning was performed only on the trained facts. For each initially correct fact, the first transition from correct to incorrect retrieval was recorded as a *fact-loss* event at the epoch in which the transition occurred. Facts that remained correctly retrieved through epoch 20 were treated as censored observations. Kaplan–Meier estimators were used to describe the time to fact loss and to compare fact retention by training status. Because the number of initially correct facts and fact-loss events was small, Cox proportional hazards models were not used to assess predictors of fact loss. Instead, we performed univariate comparisons between facts with and without fact loss for the major covariates, including baseline stochastic support.

## 3 Results

**Figure 2:**
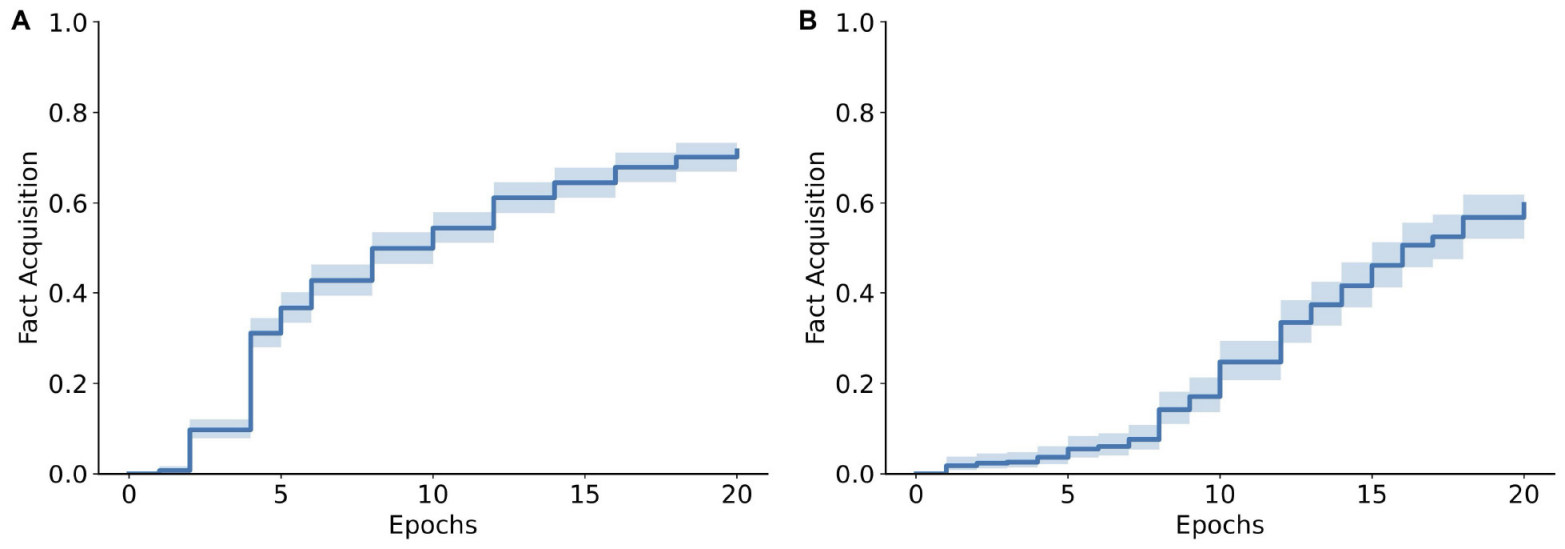
Kaplan–Meier fact-acquisition curves over 20 epochs. **(A)** shows acquisition for 800 trained HPO term-identifier pairs (cumulative acquisition 71.9% at epoch 20; shaded areas, 95% confidence intervals). **(B)** shows acquisition for 401 trained GO term-identifier pairs (cumulative acquisition 61.8% at epoch 20; shaded areas, 95% confidence intervals).

**Figure 3:**
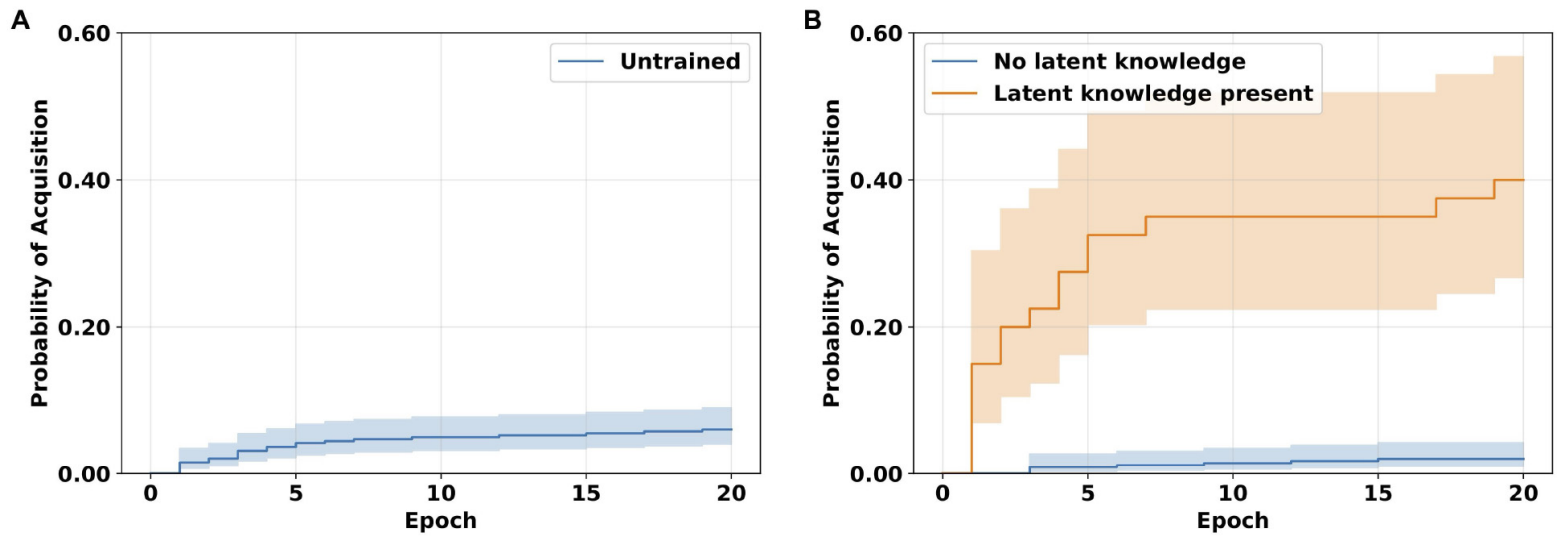
Fine-tuning acquisition of previously unacquired GO term-identification facts. **(A)** Kaplan-Meier-style cumulative acquisition curve showing the proportion of initially unacquired facts that were acquired over 20 fine-tuning epochs. Models were fine-tuned on 401 training facts and evaluated on 378 untrained facts; 23 held-out facts were acquired during fine-tuning. **(B)** Acquisition curves stratified by latent knowledge status before fine-tuning. Facts with latent knowledge present were acquired more frequently than latent-knowledge-negative facts.

At baseline (epoch 0), the pre-fine-tuned Llama-3.1-8B Instruct model correctly retrieved 1.1% (9/800) of HPO facts and 5.5% (44/802) of GO facts. After 20 epochs of fine-tuning, correct retrieval increased to 71.9% (575/800) for trained HPO facts, 61.8% (248/401) for trained GO facts, and 11.2% (45/401) for untrained GO facts (Figure 2). Among baseline-incorrect untrained GO facts, new untrained-fact acquisition occurred in 5.8% (22/378; Figure 3).

### 3.1 Latent knowledge predicts the acquisition of trained facts

**Figure 4:**
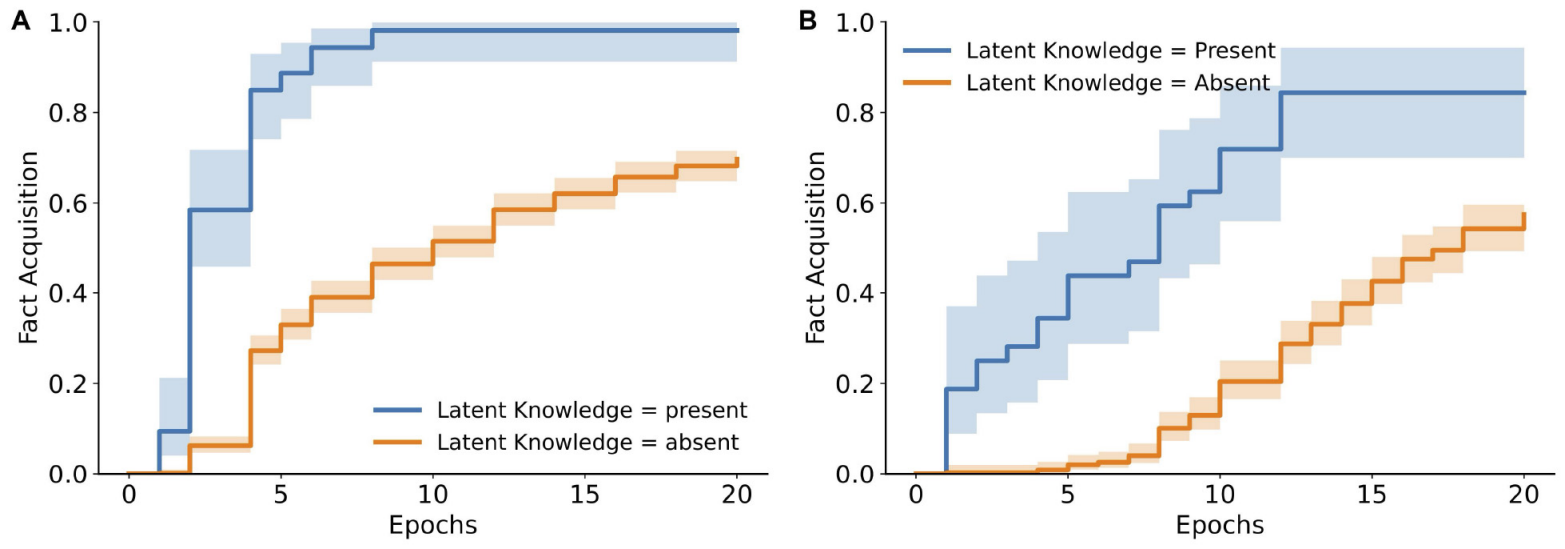
Fact-acquisition curves stratified by latent-knowledge status. **(A)** shows Kaplan–Meier curves for 800 trained HPO facts; at epoch 20, cumulative acquisition was 98.4% for latent-knowledge-positive facts and 69.7% for latent-knowledge-negative facts. **(B)** shows Kaplan–Meier curves for 401 trained GO facts, with faster acquisition for latent-knowledge-positive than latent-knowledge-negative facts.

Kaplan–Meier curves for HPO trained-fact acquisition differed markedly by latent-knowledge status (Figure 4A). Latent-knowledge-positive facts were acquired earlier and more rapidly than latent-knowledge-negative facts. Mean time to acquisition was 2.9 ± 0.4 epochs for latent-knowledge-positive HPO facts versus 11.4 ± 0.3 epochs for latent-knowledge-negative HPO facts. The curves differed significantly by the Mantel–Cox log-rank test (χ^2^ = 161.8, df = 1, and *p* <0.001).

Cox proportional hazards modeling (Table 5) identified latent-knowledge status as the strongest predictor of trained-fact acquisition for HPO facts. Latent-knowledge-positive facts were acquired more rapidly than latent-knowledge-negative facts (hazard ratio =2.60, 95% CI = 1.81–3.75, and *p* <0.001). Two external corpus-support measures were also significant positive predictors of acquisition rate: HPO annotation frequency (hazard ratio = 1.68, 95% CI = 1.53–1.84, and *p* <0.001) and HPO identifier frequency in PubMed Central (hazard ratio = 1.18, 95% CI = 1.06–1.30, and *p* = 0.002) (Kandpal et al., 2023; Ghosal et al., 2024; Christoph et al., 2025). In contrast, HPO term frequency in PubMed Central was not significantly associated with acquisition rate.

**Table 5:** Multivariable Cox proportional hazards model for acquisition of HPO facts.

Covariate	HR	95% CI	Cox *p*	Schoenfeld *p*
HPO term PMC count, log_10_ standardized	0.99	0.91–1.07	0.808	0.171
HPO ID PMC count, log_10_ standardized	1.18	1.06–1.30	0.002	0.490
HPO annotation count, log_10_ standardized	1.68	1.53–1.84	<0.001	<0.001
Latent knowledge present	2.60	1.81–3.75	<0.001	0.114

*HR, hazard ratio; CI, confidence interval; PMC, PubMed Central. HR* > *1 indicates a higher rate of trained-fact acquisition, and HR*<*1 indicates a lower rate. PubMed Central count and annotation-count variables were Laplace-smoothed and log*_10_*-transformed before model fitting; hazard ratios for these variables, therefore, correspond to a one-unit increase on the transformed scale. Latent knowledge indicates that the correct identifier was produced in at least one stochastic decoding sample before fine-tuning. Schoenfeld* *p-values are based on tests of scaled Schoenfeld residuals for time-dependent effects. A small Schoenfeld* *p-value indicates evidence against the proportional hazards assumption. The proportional hazards assumption was not rejected for HPO term PMC count, HPO ID PMC count, or latent knowledge, but was rejected for HPO annotation count*.

Tests based on Schoenfeld residuals indicated that the proportional hazards assumption was not uniformly satisfied. Among the covariates shown in Table 5, the clearest violation involved HPO annotation frequency, indicating that its association with acquisition varied across training epochs. In contrast, the latent-knowledge indicator and HPO identifier frequency did not show significant proportional-hazards violations in this model. Accordingly, hazard ratios are interpreted as summary effects over the observed fine-tuning interval, with the qualification that some corpus-support effects, particularly annotation frequency, may vary over time.

As a sensitivity analysis, we tested whether the main covariate findings persisted when acquisition was modeled as a discrete epoch-indexed binary event rather than with a Cox proportional hazards model. For HPO trained-fact acquisition, each ontology fact contributed one observation for each epoch during which it remained unacquired and therefore at risk for acquisition. The binary outcome indicated whether the fact was first retrieved correctly at that epoch. Epoch was included as a categorical predictor, allowing the baseline probability of acquisition to vary across training epochs without imposing a proportional-hazards assumption or a continuous-time event model. Results were qualitatively consistent with the Cox analysis: latent knowledge score remained the strongest predictor of acquisition, HPO annotation frequency and HPO identifier frequency in PubMed Central retained positive associations, and HPO term frequency in PubMed Central remained non-significant (Table 6). Thus, the main covariate pattern was robust to an alternative discrete-time model that treated acquisition as an epoch-indexed binary event without invoking the Cox proportional-hazards assumption.

**Table 6:** Discrete-time sensitivity analysis for HPO trained-fact acquisition.

Covariate	Odds ratio	95% CI	*p*-value
Latent knowledge score, standardized	4.31	1.96–9.52	<0.001
HPO annotation count, log_10_-transformed, standardized	2.07	1.78–2.42	<0.001
HPO ID PMC count, log_10_-transformed, standardized	1.22	1.04–1.44	0.016
HPO term PMC count, log_10_-transformed, standardized	0.94	0.84–1.06	0.309

*The person-period logistic regression model treated each epoch as a discrete time interval and modeled the discrete-time hazard of acquisition. Each term–identifier pair contributed observations until acquisition or censoring. Epoch was included as a categorical time term, allowing the baseline acquisition probability to vary by epoch; epoch coefficients are not shown. Odds ratios summarize the association between each covariate and acquisition during an epoch, conditional on remaining unacquired at the start of that epoch. Unlike the Cox proportional hazards model, this approach does not require a continuous-time proportional-hazards assumption, although covariate effects are assumed constant on the log-odds scale unless covariate-by-epoch interactions are included. Continuous covariates were transformed and standardized as in the Cox proportional hazards models*.

### 3.2 Latent knowledge predicts the acquisition of untrained facts

We next asked whether fine-tuning could induce *untrained-fact acquisition* for GO facts withheld from the fine-tuning set. This analysis included 378 withheld GO facts that were incorrect at baseline.

Untrained-fact acquisition was uncommon. Over 20 epochs, 22 of 378 untrained facts (5.8%) became correct. Among acquired untrained facts, the median time to acquisition was three epochs (interquartile range 1.5–6.5), indicating that successful acquisition occurred early in fine-tuning rather than gradually accumulating over later epochs.

A Cox proportional hazards model fitted to the untrained GO facts identified latent-knowledge status and identifier corpus support as significant predictors of acquisition (concordance = 0.87; log-likelihood ratio test, *p* <0.001; Table 7). Latent-knowledge-positive untrained facts were more likely to be acquired than latent-knowledge-negative untrained facts (hazard ratio = 14.17, 95% CI = 4.60–43.60, and *p* <0.001). Higher GO identifier frequency in PubMed Central was also associated with increased acquisition rate (hazard ratio = 3.33, 95% CI = 1.30–8.53, and *p* = 0.01). In contrast, GO annotation counts and GO term frequencies in PubMed Central were not significant after controlling for these effects. Schoenfeld residual tests did not indicate violation of the proportional hazards assumption for the covariates in this model.

**Table 7:** Multivariable Cox proportional hazards model for GO untrained-fact acquisition.

Covariate	HR	95% CI	Cox *p*	Schoenfeld *p*
Latent knowledge present	14.17	4.60–43.60	<0.01	0.342
GO annotation count, log_10_ standardized	0.80	0.44–1.47	0.47	0.097
GO ID PMC count, log_10_ standardized	3.33	1.30–8.53	0.01	0.823
GO term PMC count, log_10_ standardized	0.79	0.55–1.12	0.19	0.096

*HR, hazard ratio; CI, confidence interval. The analysis included 378 untrained GO facts that were incorrect at baseline; 22 acquisition events occurred and 356 observations were censored. Schoenfeld* *p-values are based on tests of scaled Schoenfeld residuals for time-dependent effects; small* *p-values indicate evidence against the proportional hazards assumption*.

Taken together, these results suggest that fine-tuning can occasionally induce acquisition of GO facts that were withheld from training, but such acquisition was uncommon. It occurred primarily for latent-knowledge-positive untrained facts and for identifiers that were more frequent in PubMed Central, underscoring the role of pretraining-derived parametric structure in determining when untrained-fact acquisition is possible.

### 3.3 Fact loss

**Figure 5:**
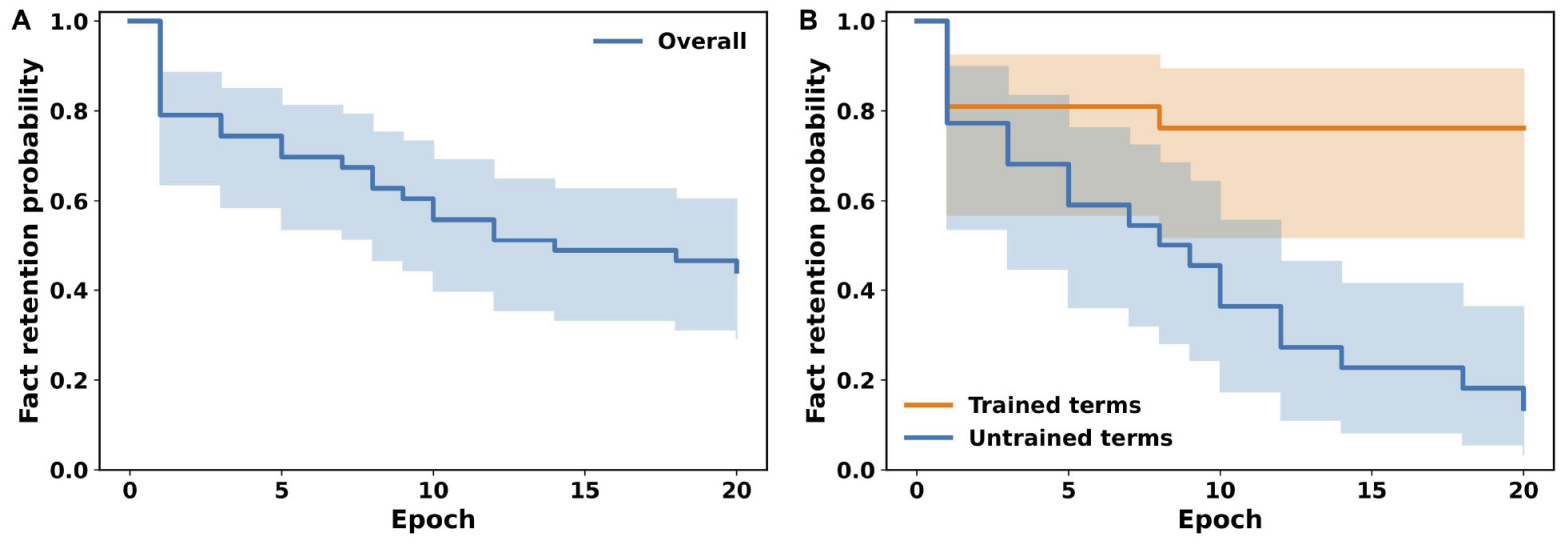
Fact loss among GO facts correct at baseline. **(A)** shows the Kaplan–Meier curve for fact loss among 44 GO facts that were correctly retrieved at epoch 0, where fact loss was defined as the first transition from correct to incorrect retrieval across epochs 1–20 (estimated risk of loss 54.5% at epoch 20; shaded area, 95% confidence interval). **(B)** shows Kaplan–Meier fact-loss curves stratified by training status, with lower fact loss among trained than untrained facts (Mantel-Cox log-rank χ = 14.5, *p* <0.001).

To evaluate whether fine-tuning disrupted previously correct fact retrieval, we analyzed the 44 GO facts that were correctly retrieved at baseline (epoch 0). During fine-tuning, 24 of these 44 facts (54.5%) were lost; that is, the model transitioned from producing the correct identifier at baseline to producing an incorrect identifier at one or more subsequent epochs (Figure 5A).

When these facts were stratified by training status, fact retention was higher among trained facts than among withheld facts (Figure 5B). The Kaplan–Meier fact-retention curves differed significantly by the Mantel–Cox log-rank test (χ^2^ = 14.5, *p* <0.001).

Because the number of initially correct facts was small, we did not fit a Cox proportional hazards model for fact loss. Instead, we performed univariate comparisons between facts with and without fact loss (Table 3). Facts with fact loss were less likely to have been included in the fine-tuning set and had lower baseline stochastic support. These findings suggest that fact loss during fine-tuning was common and was not random: facts withheld from the fine-tuning set were more vulnerable to loss, whereas trained facts were more likely to be retained. Higher baseline stochastic support for the correct identifier may also have been associated with greater retention of initially correct facts.

## 4 Discussion

This study provides a framework for viewing fact acquisition during the fine-tuning of large language models as a time-to-event process, in which the key quantities of interest are the rates and timing of fact acquisition and fact loss rather than solely the final binary outcomes. Using facts from the Human Phenotype Ontology and the Gene Ontology as structured sets of learnable facts (Hier et al., 2025b; Pericharla et al., 2026), we characterized how rapidly new facts were acquired during fine-tuning and how these rates depended on properties of the individual facts and on latent parametric support that was detectable through stochastic decoding prior to fine-tuning.

Binary accuracy metrics can obscure important differences in model behavior during fine-tuning: two fine-tuned models with similar final accuracies may reach that state along different learning trajectories. Thus, we applied Cox proportional hazards models to identify predictors of fact acquisition rate within a time-to-event framework. The strongest predictor was *latent knowledge detectable before fine-tuning*: facts that were not retrieved under deterministic decoding but were recoverable under stochastic decoding were acquired more rapidly than facts without detectable latent knowledge (Gekhman et al., 2024, 2025). Among HPO facts, latent-knowledge-positive facts were acquired more completely than latent-knowledge-negative facts (100% vs. 60%), and the importance of latent knowledge as a predictor of fact acquisition was confirmed by both the Kaplan–Meier plots (Figure 4) and the Cox proportional hazards model (Table 5).

The results also highlight *corpus-based fact support* as a second, independent determinant of fine-tuning success. In the HPO Cox model, higher ontology annotation counts and higher HPO identifier frequencies in PubMed Central were associated with faster trained-fact acquisition (hazard ratios 1.68 and 1.18, respectively; Table 5). For withheld GO facts, higher GO identifier frequency in PubMed Central similarly predicted greater untrained-fact acquisition (hazard ratio 3.33; Table 7). These corpus-based measures of fact support parallel recent findings that fine-tuning is more effective when applied to facts that are already well represented in the pretraining corpus. Ghosal et al. (Ghosal et al., 2024) showed that fine-tuning on more popular, well-encoded facts improves factual QA performance even for less-popular test facts, whereas fine-tuning directly on poorly encoded long-tail facts can amplify shortcut behavior and harm factuality.

Latent knowledge should not be interpreted as an intrinsic property of an ontology fact. Rather, it is a property of the relationship between a specific pretrained model and a specific ontology fact. The same ontology fact may be latent-knowledge-positive in one model and latent-knowledge-negative in another, depending on the model's pretraining corpus, architecture, scale, and parameterization. This model-specificity is important because fine-tuning may be most effective when it amplifies weak but already present associations, and less effective when the association is absent or poorly represented in the pretrained model (Hier et al., 2025b). Conversely, facts that are already correctly retrieved at baseline provide little opportunity for measurable acquisition and may still be vulnerable to loss during fine-tuning (Figure 5). Thus, the greatest observable benefit of fine-tuning may occur for facts occupying an intermediate zone: represented well enough to be reinforced, but not yet reliably retrieved.

Although we did not examine the model's internal representations or output distributions directly, our findings are consistent with the idea that latent knowledge constrains the set of plausible identifiers during fine-tuning. For latent-knowledge-positive facts, the pretrained model already appears to assign some recoverable probability mass to the correct term–identifier association. Fine-tuning may strengthen an existing weak association rather than establish a new association. In this sense, detectable latent knowledge gives the model a *head start* in acquiring a new fact.

This interpretation could help explain faster initial acquisition for HPO and GO latent-knowledge-positive facts (Figure 4). In contrast, latent-knowledge-negative facts may begin with less-informative next-token probabilities distributed across many possible identifiers and may require more gradual gradient updates before the correct association becomes retrievable (Speicher et al., 2024). Under this interpretation, which remains speculative, fact acquisition is driven by a redistribution of next-token probability mass, with fine-tuning shifting probability toward the correct identifier (Chang et al., 2024). Other mechanisms—such as embedding realignment or changes in decision boundaries—may also contribute, but we did not examine them directly here (Yavas et al., 2025; Rajaee and Pilehvar, 2021; Zhao et al., 2024).

The experiments with Gene Ontology facts extended these findings beyond HPO and allowed us to examine *untrained-fact acquisition* during fine-tuning. For GO facts withheld from training, untrained-fact acquisition was uncommon: across 20 epochs, 5.8% (22 of 378) baseline-incorrect untrained facts changed from incorrect to correct (Figure 3). These events were enriched among latent-knowledge-positive untrained facts. In the Cox model (Table 7), detectable latent knowledge before fine-tuning increased the acquisition rate by more than an order of magnitude (hazard ratio = 14.17), and higher GO identifier counts in PubMed Central provided an additional, smaller effect (hazard ratio = 3.33). Latent-knowledge-negative untrained facts showed nearly flat cumulative acquisition curves, whereas the small subset of latent-knowledge-positive untrained facts had a higher probability of becoming correct during fine-tuning. Thus, acquisition of untrained facts was concentrated among GO facts for which the base model already appeared to have weak but detectable access to the correct association. This pattern is consistent with Lu et al. (2024), who reported that LLMs trained on collections of atomic facts can sometimes acquire untrained fact triples, particularly when query–response pairs share exploitable correlation structure.

We also observed that fine-tuning carried a measurable cost in the form of fact loss (Pletenev et al., 2025), echoing prior work on finetuning-induced degradation of factual knowledge (Kazemi et al., 2023). Among GO facts that were correct at baseline (epoch 0), 54.5% (24/44) became incorrect at least once during the 20-epoch fine-tuning run. Fact loss affected both trained and withheld facts, but occurred more frequently and earlier among withheld facts (Figure 5). Kaplan–Meier fact-retention curves showed a steeper decline in the probability of remaining correct for withheld facts, and the Mantel–Cox log-rank test confirmed a significant difference between trained and withheld groups. Because the number of initially correct GO facts was small, we did not fit Cox proportional hazards models for fact loss. Instead, we performed univariate comparisons between facts with and without fact loss. Facts with fact loss were less likely to have been included in the training set and had lower baseline stochastic support, suggesting that direct training exposure and stronger pre-existing parametric support were associated with greater retention of initially correct mappings (Table 3).

Taken together, these results suggest complementary roles for latent knowledge and training exposure during fine-tuning. Latent knowledge in the base model identified ontology facts that were more readily acquired, whereas continued training exposure was associated with greater retention of initially correct mappings. This pattern is consistent with prior reports that fine-tuning can improve performance in targeted domains while eroding weakly supported knowledge (Pletenev et al., 2025).

### 4.1 Implications for biomedical informatics

Although our experiments focused on HPO and GO facts, the same framework applies to any ontology-grounded fact with a verifiable term–code association, including SNOMED CT, RxNorm, LOINC, and ICD codes. Time-to-event analysis adds information beyond final binary accuracy by distinguishing facts that are acquired rapidly, acquired only after extended fine-tuning, remain unacquired, or are later lost.

This distinction is important for biomedical informatics because terminology retrieval is not merely a benchmark task. Incorrect or unstable code retrieval can affect cohort identification, clinical decision support, quality measurement, pharmacovigilance, billing, and downstream analytics. Some studies have suggested that AI systems that perform well on structured benchmarks may perform poorly in real-world clinical settings (Hager et al., 2024). This framework could be useful in identifying facts that are held robustly by large language models and which are fragile and subject to loss. This could have significant implications for use of these models in clinical settings.

Uncertainty-aware evaluation provides a complementary perspective (Xie et al., 2025; Seoni et al., 2023). Xie et al. describe several approaches to estimating uncertainty in LLM outputs, including verbalized confidence, consistency across repeated generations, and token-probability-based confidence measures. Their work treats token probabilities as proxies for internal model confidence, including response confidence derived from generated-token probabilities and evaluation confidence derived from the probability assigned to an evaluator's judgment token (Xie et al., 2025). In medical-domain evaluation, verbalized confidence has been used to relate answer correctness to self-expressed model confidence, although such estimates may remain poorly calibrated and overconfident (Madrid et al., 2025). Although, we did not assess model verbalized confidence in this study, our stochastic support probe addresses a related question: whether the correct ontology identifier is recoverable from the model's output distribution under stochastic decoding when it is not produced under deterministic decoding.

### 4.2 Relation to existing literature on fact memorization

The literature on factual memorization in LLMs is broad, but fewer studies have directly measured the epoch-by-epoch timing of individual fact acquisition during fine-tuning. Related work has examined factual knowledge injection, long-tail factual recall, factual knowledge extraction after fine-tuning, gradual learning of partially mastered knowledge, and the risk that new factual fine-tuning may degrade pre-existing knowledge (Kazemi et al., 2023; Ovadia et al., 2024; Gekhman et al., 2024; Lu et al., 2024; Li et al., 2024; Zucchet et al., 2025; Hu et al., 2023). The present study contributes to this narrower rate-based literature by treating each ontology term–identifier mapping as a discrete factual unit and recording the epoch at which that fact is acquired or lost.

Prior work also suggests that factual acquisition is strongly shaped by fact popularity, corpus support, and pre-existing parametric salience. Frequent or popular facts are more likely to be encoded and retrieved during pretraining, whereas long-tail facts are harder to learn and recall (Kandpal et al., 2023; Chang et al., 2024; Lu et al., 2024; Mallen et al., 2023). Related studies have shown that co-occurrence structure, entity popularity, and prior token probabilities can influence whether facts are retrieved, generalized, overwritten, or forgotten (Kang and Choi, 2023; Sun et al., 2025). Our findings extend this line of work by showing that latent support detectable by stochastic decoding before fine-tuning predicts not only whether an ontology fact is eventually retrieved, but also how rapidly it is acquired. Exploratory fact-loss analyses further suggest that stronger baseline parametric support may be associated with greater retention of previously correct ontology facts.

### 4.3 Limitations

This study has several limitations. We operationalized factual acquisition as learning the identifier associated with a biomedical ontology term. Although other formulations of fact acquisition are possible, the term–identifier paradigm has important methodological advantages: correctness can be assessed unambiguously, all facts share a common structural form, and large-scale annotation frequencies provide empirically grounded proxies of fact popularity in biomedical corpora. Nevertheless, it remains uncertain whether findings from this paradigm will generalize to other forms of factual acquisition.

The present study should be interpreted as a proof-of-concept demonstration of a time-to-event framework for ontology fact acquisition and loss, rather than as a comprehensive evaluation across model families, fine-tuning configurations, prompt formulations, or ontology domains. The fine-tuning experiments varied the number of training epochs from 0 to 20 using datasets of 800 HPO facts and 802 GO facts. Other hyperparameters, including learning rate, batch size, LoRA rank, scheduler settings, and optimization strategy, were not systematically explored. In performance-optimization studies, extensive hyperparameter exploration is often central to the modeling objective (Nejadshamsi et al., 2025a,b); in contrast, our goal was methodological: to evaluate whether ontology fact acquisition and loss could be represented as time-to-event outcomes under a fixed, reproducible fine-tuning configuration. We also used a standardized evaluation prompt to preserve comparability across epochs. A limited base-model prompt-sensitivity analysis was performed, but we did not evaluate prompt sensitivity across all fine-tuned epoch checkpoints. Future work should test whether the same fact-level predictors of acquisition and loss remain informative across additional base models, LoRA configurations, learning schedules, prompt formulations, and ontology or terminology systems, including both biomedical and non-biomedical domains.

The survival analyses were epoch-indexed rather than continuous-time analyses. Training epoch has no direct real-world time equivalent, such as days or months, and multiple facts could be acquired or lost at the same epoch. Kaplan–Meier estimators were therefore used descriptively to summarize the probability of remaining unacquired or retained across discrete fine-tuning epochs. Cox proportional hazards models were used to identify covariates associated with trained-fact acquisition and untrained-fact acquisition, with tied event times handled using Efron's approximation. Because the event process was observed only at discrete epochs, we interpret Cox hazard ratios as summary associations over the observed fine-tuning interval rather than as estimates of a continuous-time biological or clinical hazard process. Furthermore, we considered only the first time a fact was acquired or lost as a measurable *event*. We did not evaluate *fact instability* during fine-tuning.

Schoenfeld residual diagnostics were used to assess the proportional hazards assumption for the Cox models. The proportional hazards assumption was not rejected for most covariates, although it was not uniformly satisfied across all models and predictors. Accordingly, hazard ratios should not be interpreted as strictly time-invariant effects in every case.

When we assessed fact loss during fine-tuning, the number of initially correct ontology facts was small. Therefore, we did not fit Cox proportional hazards models for fact loss. Instead, fact loss was summarized using Kaplan–Meier fact-retention curves, log-rank testing, and univariate comparisons between facts that were lost and facts that were retained during fine-tuning. Accordingly, the fact-loss findings should be interpreted as exploratory and hypothesis-generating. Nonetheless, these exploratory findings suggest that continued exposure during fine-tuning and stronger baseline parametric support may be associated with greater retention of previously correct facts.

These analyses focused on changes in model accuracy and acquisition timing, rather than on the internal mechanisms underlying these changes. We did not directly examine shifts in logits, parameter updates, or representational geometry during fine-tuning. As a result, the study characterizes what facts are acquired and when, but not the mechanisms by which fine-tuning alters internal representations (Ghosal et al., 2024; Yavas et al., 2025; Rajaee and Pilehvar, 2021; Zhao et al., 2024; Feng et al., 2024). Integrating mechanistic explanations with the proposed time-to-event acquisition framework is an important direction for future work.

We excluded approximately 40% of HPO terms and 54% of GO terms that lacked curated biomedical literature annotations (Hier et al., 2025a). Long-tail terms from sparsely represented regions of the ontology have proven difficult for base LLMs to retrieve from parametric knowledge alone. Consequently, the observed acquisition rates depended on a sampled subset of ontology facts and likely represent performance closer to the upper bound than the lower bound of what would be expected across the full ontologies. The results should therefore be interpreted as applying to ontology facts with at least minimal representation in curated biomedical resources, rather than to the sparsest regions of HPO or GO. In addition, our experiments used a local Llama-3.1-8B Instruct model evaluated without retrieval augmentation, tool use, web search, or access to external terminology services. The findings characterize acquisition and loss of facts retrieved from parametric model knowledge, and should not be assumed to apply to frontier foundation models that can retrieve or verify facts using external non-parametric fact sources.

Finally, the proposed framework assumes that acquisition can be scored as a discrete event, such as the first epoch at which a model produces the correct ontology fact. This assumption may not hold for tasks with gradual, partial, or multidimensional learning signals. The approach is also computationally demanding, requiring repeated stochastic-decoding queries to estimate stochastic support and detect latent knowledge (Gekhman et al., 2025), as well as full test-set evaluation of fact acquisition after each fine-tuning epoch.

## 5 Conclusions

We introduce a time-to-event, survival-analysis framework for assessing factual acquisition during model fine-tuning at the level of individual ontology facts. This approach quantifies both *rate* and *success* of fact acquisition, providing a richer description of factual learning than final accuracy alone. Using ontology facts from HPO and GO as structured test cases, we modeled three outcomes: trained-fact acquisition, untrained-fact acquisition, and fact loss. We use these operational terms rather than more common labels such as memorization, generalization, and forgetting, because those labels can imply underlying mechanisms that were not directly tested in this study.

Our HPO experiments show that latent-knowledge-positive facts were acquired more rapidly and more completely than latent-knowledge-negative facts. Our GO experiments show that latent-knowledge-positive untrained facts were more likely than latent-knowledge-negative untrained facts to become acquired after fine-tuning, although untrained-fact acquisition remained uncommon. In the fact-loss analysis, initially correct facts with higher baseline stochastic support were more resistant to loss, and continued exposure to the facts during fine-tuning appeared additionally protective.

Taken together, these findings suggest that pretraining-derived parametric structure increases the efficiency and stability with which factual associations are acquired and retained during fine-tuning. Time-to-event modeling revealed whether facts were acquired, when as well as whether they were acquired despite being withheld from training or lost during fine-tuning. The findings also suggest a potential protective effect of continued training on known facts.

The proposed rate-based framework can help guide future work on fine-tuning strategies, curriculum design, and evaluations of learning efficiency and stability in large language models, particularly in ontology-grounded biomedical applications. These results contribute to the unresolved question of whether fact-injection curricula should include new facts as well as rehearsing previously known facts.

## Data Availability

The datasets presented in this study can be found in online repositories. The names of the repository/repositories and accession number(s) can be found below: Zenodo: 10.5281/zenodo.17762927.
